# Prevalence and Predictors of Dermatophyte Infections Among Primary School Children in Ilemela, Mwanza, Tanzania

**DOI:** 10.24248/EAHRJ-D-18-00033

**Published:** 2019-07-30

**Authors:** Martha F Mushi, Editha Jonathan, Mariam M Mirambo, Stephen E Mshana

**Affiliations:** a Microbiology and Immunology Department, Catholic University of Health and Allied Science, Weill Bugando School of Medicine, Mwanza, Tanzania; b School of Public Health, Catholic University of Health and Allied Science, Mwanza, Tanzania

## Abstract

**Background:**

Dermatophytes are highly contagious organisms of public health importance, particularly among primary school children in the resource-limited settings with a prevalence of 10% to 20% in East Africa. Here, we report the prevalence and associated factors of dermatophyte infections among primary school children in Ilemela, Mwanza - Tanzania.

**Methods:**

A cross-sectional study was conducted involving 323 children aged between 4 and 10 years from 10 randomly selected primary schools. The study was conducted between July 2017 and September 2017. Pretested interviewer-administered semi-structured questionnaire was used to collect relevant social-demographic information followed by clinical examination to establish the diagnosis of dermatophyte infections. Data were analysed using Stata version 13.

**Results:**

The mean age of the study participants was 7.63±1.27 years, with the slightl majority (n=183, 56.7%) of participants being girls. The majority (n=277, 70.3%) of the study participants were from public schools. A total of 299 (92.6%) children reported using tap water at home. Using clinical diagnosis, 94 (29.1%) children had dermatophyte infections with 92 (97.9%) of them having tinea capitis. By multivariate logistic regression analysis: being a boy (odds ratio [OR] 1.98; 95% confidence interval [CI], 1.22 to 3.22; *P*=.01); using lake, river, or well water (OR 3.18; 95% CI, 1.36 to 7.38; *P*<.01); playing in a dusty environment (OR 2.65; 95% CI, 1.28 to 5.47; *P*<.01); playing with animals (OR 2.13; 95% CI, 1.28 to 3.56; *P*<.003); and having family members with dermatophyte infections (OR 10.56; 95% CI, 4.57 to 24.41; *P*<.001) predicted dermatophyte infections.

**Conclusion:**

The prevalence of dermatophyte infection is high in the study population and is associated with poor hygiene. Improved hygiene will reduce the prevalence of dermatophyte infections among primary school children in low-income countries. Further studies to identify the species and susceptibility patterns of these dermatophytes are recommended to establish empirical treatment guidelines.

## INTRODUCTION

Dermatophyte infections are common superficial fungal infections of public health importance in areas with poor personal and domestic hygiene.^1^ Due to the presence of a low amount of inhibitory fatty acids in the skin of prepubertal children, dermatophyte infections tend to occur more in this age group and mainly affects scalp and skin.^2,3^ In East Africa, the prevalence of dermatophyte infections has been reported to range from 10% to 20% among primary school-aged children^4-6^ with limited information from rural areas. Tinea capitis was reported to be endemic in Africa with more than 20 million people affected in the past 4 decades.^7^

Dermatophyte infection is of public health concern due to its contagious nature, as it has been found to be easily transmitted through close skin-to-skin contact with an infected person, sharing of combs and clothes, and playing with domestic animals.^4^ Asymptomatic carriers and the seasonal nature of the disease significantly contribute to dermatophyte transmission between close contacts.^8,9^ The epidemiological distributions of dermatophyte infections have geographical and seasonal variations, depending on the migration of people and climatic conditions. Poor living conditions (overcrowding, poor sanitation, low water supplyn and low socioeconomic status), close contact with infected children, and playing with domestic animals have been found to predispose prepubertal children to dermatophyte infections.^4,9^

Dermatophyte infections can lead to social stigma, resulting in psychosocial trauma to the affected children. The irritation of the affected area due to inflammatory reactions affect children's concentration in class, potentially leading to poor performance and school dropout.^4^ Additionally, ulceration of the affected area increases susceptibility to secondary bacterial infections.

Despite the physical and psychosocial sequelae that can be brought about by dermatophyte infections, this group of diseases remains neglected in low-income countries. This is partially because most of these fungal infections are benign^10^; as a result, robust epidemiological data are scarce. The present study provides data on the prevalence and factors associated with dermatophyte infections among primary school children in Ilemela, Mwanza, Tanzania. These data are important for identifying the high-risk groups among these children and will inform appropriate preventive strategies.

## METHODS

### Study Design and Study Area

This was a cross-sectional study conducted from July 2017 to September 2017 in primary schools in Ilemela District, Mwanza, Tanzania. According to the national bureau of statistics of Tanzania, Mwanza is the second most densely populated region after Dar es Salaam, with approximately 1,294,761 children aged 0 to 14 years.^11^ Mwanza city has two administrative districts, namely Nyamagana and Ilemela. We chose Ilemela District out of convenience. The district has 74 primary schools and 46 health centres (17 public and 29 private). Participating schools were selected at random.

### Sample Size, Sampling, and Inclusion Criteria

The representative target population (323 primary school children) was estimated using Cochran's equation,^12^ assuming a prevalence of 30.4% based on previous study findings from Dar es salaam, Tanzania.^6^ Ten schools were randomly selected. Pupils were randomly chosen until the desired sample size and distribution across schools was reached.

### Data Collection

A trained nurse used a semistructured, pretested, interviewer-administered questionnaire to collect sociodemographic and clinical data. If a child failed to provide the required information, a checklist was provided to the child's parents to fill. Physical assessment to detect skin lesions suggestive of dermatophyte infection was done. Tinea capitis was defined by the presence of dull, grey, circular patches of alopecia, which is scaling and itching, while tinea corporis was defined by the presence of annular lesions with a clearing, scaly centre surrounded by a red, advancing border that was either dry or vesicular.^13^ Physical assessment and questionnaire administration took about 30 minutes.

### Data Management

The data were entered and cleaned using Microsoft Excel (Microsoft Corp., Redmond, WA, USA) and analysed using Stata version 13 (StataCorp, College Station, TX, USA). Continuous variables, such as age and family size, were summarised using means and standard deviations. Categorical variables were described as proportions and percentages. A stepwise logistic regression analysis was employed to determine factors associated with dermatophyte infection. All factors which were statistically significant on univariate analysis were subjected to multivariate logistic regression analysis. The statistical significance was set at the 95% confidence level, wherein *P*<.05 was considered statistically significant.

### Ethical Considerations

The study protocol was reviewed and approved by the Joint Catholic University of Health and Allied Sciences/Bugando Medical Centre (CUHAS/BMC) Research Ethics and Eeview Committee (CREC)) certificate no: CREC/274/2017). Students were given information regarding the study and provided with the informed consent form to give to their parents. Children were included if they provided assent and their parents consented.

## RESULTS

### Demographic Characteristic of Study Participants

A total of 323 primary school children were involved in the study, including 183 (56.7%) girls. The mean age of the study participants was 7.6±1.2 years, and the mean family size was 5.5±2 people. The majority of the children were from public schools (n=225, 69.7%), and 299 (92.6%) children reported using clean tap water at home (Table 1).

**TABLE 1.** Sociodemographic Characteristics (N=323)

Variable	n (%)
**Sex**	
Girls	183 (56.7)
Boys	140 (43.3)
**Age, years±standard deviation**	7.63±1.27
**School ownership**	
Private	98 (30.3)
Government	225 (69.7)
**Playground surface**	
Dust	258 (79.9)
Cement/grass	65 (20.1)
**Plays with domestic animals**	
Yes	96 (29.7)
No	227 (70.3)
**Family size, mean±standard deviation**	5.532±1.967
**Mode of family**	
Both parents	268 (83)
Single parent	55 (17)
**Source of water**	
Clean tap water	299 (92.6)
Lake/well/river	24 (7.4)
**Towel sharing**^a^	
Yes	59 (22.3)
No	205 (77.6)
**Comb sharing**	
Yes	169 (52.3)
No	154 (47.7)
**Friends with dermatophytosis**	
Yes	48 (14.9)
No	275 (85.1)
**Family member with dermatophytosis**	
Yes	289 (89.5)
No	34 (10.5)

a 59 students did not use towels

### Dermatophyte Infections

A total of 94 (29.1%) children were clinically diagnosed with dermatophyte infections. The majority (n=92, 97.9%) had tinea capitis, with only 2 (2.1%) children diagnosed with tinea corporis. Of 140 boys, 52 (37.1%) had dermatophyte infection compared with 42 (22.2%) of 183 girls children (X^2^= 7.7; *P*<.01). There was a significantly higher prevalence of dermatophyte infection among children attending public schools compared with private schools (n=69, 35.8% vs n=14, 14.3%, respectively; *P* <.001) (Table 2).

**TABLE 2.** Factors Associated With Dermatophyte Infections Among Primary School Children (N=323)

	Variable	Dermatophytes		X^2^	*P* Value
		Yes	No		
**Sex**	Girls	42 (22.2%)	141 (77.1%)	7.7430	.005
	Boys	52 (37.1%)	88 (62.9%)		
**School ownership**	Private	14 (14.3%)	84 (85.7%)	14.6899	.001
	Government	69 (35.8%)	124 64.3%)		
**Family type**	Single parents	13 (36.1%)	23 (63.9%)	1.3468	.25
	Both Parents	72 (26.9%)	196 (73.1%)		
**Source of water**	Tape	81(27.1%)	218 (72.1%)	7.8941	.005
	Lake/well/river	13(54.2%)	11 (45.8%)		
**Playground surface at school**	Not dusty	10 (15.4%)	55 (84.6%)	7.4215	.01
	Dusty	84 (32.6%)	174 (67.4%)		
**Plays with pets**	No	55 (24.2%)	172 (75.8%)	8.7904	.003
	Yes	39 (40.6%)	57 (59.4%)		
**Friend with dermatophytosis**	No	67 (24.4%)	208 (75.6%)	20.1383	.001
	Yes	27 (56.3%)	21 (43.8%)		
**Family member with dermatophytosis**	No	68 (23.5%)	221 (76.5%)	41.3241	.001
	Yes	26 (76.5%)	8 (23.5%)		

### Predictors of Dermatophyte Infections

Multivariable logistic regression analysis revealed that male gender (adjusted odds ratio [AOR] 2.04; 95% confidence interval [CI], 1.07 to 3.90; *P*<.029), attending public school (AOR 2.27; 95% CI, 1.04 to 4.98; *P*<.039), not changing uniform at least weekly (AOR 4.56; 95% CI, 1.11 to 18.71, *P*<.035), playing with domestic animals (AOR 2.04; 95% CI, 1.3 to 4.02; *P*<.030), and sharing of bed sheets (AOR 6.35; 95% CI, 3.32 to 12.15; *P*<.001) were significant predictors of dermatophyte infections among the participating primary school children (Table 3).

**TABLE 3.** Multivariate Logistic Regression Analysis of Predictors of Dermatophytes

Variable	OR	95% CI	*P* Value	AOR	95% CI	*P* Value
**Sex**						
Female	1			1		
Male	1.98	1.22–3.22	.01	2.04	1.07–3.90	.029
**Type of school**						
Private	1					
Government	3.34	1.76–6.32	.001	2.27	1.04–4.98	.08
**Type of family**						
Single mother/guardian	1					
Both parents	0.64	0.31–1.35	.25	-----	-----	--------
**Source of water**						
Tap	1					
Lake/river/well	3.18	1.36–7.38	.01	2.49	0.87–7.10	.08
**Reported towel sharing**						
No	1					
Yes	1.25	0.65–2.39	.49	-----	-----	------
**Frequency of changing school uniform within a week**						
Twice	1					
Once	1.33	0.81–2.20	.25	1.22	0.62–2.39	.55
None	2.97	0.97–9.02	.05	4.56	1.11–18.71	.035
**Playground surface**						
Not dusty	1					
Dusty	2.65	1.28–5.47	.01	1.71	0.71–4.13	.23
**Reported playing with animals**						
No	1					
Yes	2.13	1.28–3.56	.003	2.04	1.03–4.02	.038
**Reported having family members with dermatophytes**						
No	1					
Yes	10.56	4.57–24.41	.001	7.66	2.75–21.26	.0011
**Reported sharing of bed sheets**						
No	1					
Yes	5.62	3.34–9.45	.001	6.35	3.32–12.15	.001

Abbreviations: AOR, adjusted odds ratio; CI, confidence interval; OR, odds ratio

## DISCUSSION

Dermatophyte infections are common and remain an important public health problem among primary school children in resource-limited settings, including Tanzania. This was evident in the present study whereby about one-third of children had dermatophyte infections. The observed prevalence was similar to the 30.4% and 33.3% reported among primary school children in Dar es Salaam and Kenya, respectively.^2,6^ Nevertheless, the reported prevalence was lower than what was reported in other previous studies conducted in Africa – 59% in Ethiopia^14^ and 81.2% in Kenya, for example^15^. The observed differences could be explained by the differences in the study populations; in the previous studies, the majority of children were from slums and public schools, while the present study involved both public and private schools.

As has been previously observed elsewhere,^15-18^ the prevalence of dermatophyte infections in the present study was significantly higher among boys compared with girls. The higher prevalence among boys has been attributed to several factors, such as poorer personal hygiene, sharing of combs, playing in dust, sharing of towels, and interacting and playing with friends without considering personal hygiene.^15-18^ It should be noted that studies conducted in Nigeria and Egypt reported significantly higher prevalences of dermatophyte infections among girls compared with boys.^19,20^

As observed in previously,^9^ tinea capitis was the predominant dermatophyte infection detected in this study. Male gender, having family member with a dermatophyte infection, not using tap water, and sharing combs significantly predicted tinea capitis. Similar observations have been reported from previous studies^9,15,18,21,22^ investigating primary school children.

This work was limited by a lack of capacity for fungal culture; dermatophyte infection was solely a clinical diagnosis, and this might have inflated the prevalence findings.

## CONCLUSION

A high proportion of children with poor hygiene had tinea capitis. Health education tailored to boys regarding personal hygiene will significantly reduce the burden of dermatophyte infections. Further studies should be conducted to identify the patterns and distributions of the dermatophyte species that cause these common infections in children.
